# Supplementary material for: Democratized single-cell proteomics resolves cell state heterogeneity in skin tumors
Source: Life Sci Alliance. 2026 Jun 29;9(9):e202603759. doi: 10.26508/lsa.202603759 (PMC13315484; doi:10.26508/lsa.202603759)
Supplement: Supplementary file 3 [file LSA-2026-03759_TableS1.docx]

**Supplementary table 1. Patient samples used in the study.**

| **ID** | **Age** | **Gender** | **Experiment** | ***CYLD* Genotype** |
| --- | --- | --- | --- | --- |
| Sample 1 | 52 | F | Single cell proteomics (pilot) | c.2339delT |
| Sample 2 | 54 | F | Single cell proteomics | c.2460delC |
