# Supplementary material for: Democratized single-cell proteomics resolves cell state heterogeneity in skin tumors
Source: Life Sci Alliance. 2026 Jun 29;9(9):e202603759. doi: 10.26508/lsa.202603759 (PMC13315484; doi:10.26508/lsa.202603759)
Supplement: Supplementary file 4 [file LSA-2026-03759_TableS2.docx]

**Supplementary table 2. Bruker timsTOF HT diaPASEF acquisition scheme for single cell proteomics.**

| **#MS Type** | **Cycle Id** | **Start IM [1/K0]** | **End IM [1/K0]** | **Start Mass [*m/z*]** | **End Mass [*m/z*]** | **CE [eV]** |
| --- | --- | --- | --- | --- | --- | --- |
| MS1 | 0 | - | - | - | - | - |
| PASEF | 1 | 0.7 | 0.93 | 300.17 | 478.78 | - |
| PASEF | 1 | 0.93 | 1.2 | 654.66 | 714.9 | - |
| PASEF | 2 | 0.7 | 0.97 | 478.78 | 544.28 | - |
| PASEF | 2 | 0.97 | 1.2 | 714.9 | 776.98 | - |
| PASEF | 3 | 0.7 | 1.01 | 544.28 | 601.33 | - |
| PASEF | 3 | 1.01 | 1.2 | 776.98 | 848.92 | - |
| PASEF | 4 | 0.7 | 1.06 | 601.33 | 654.66 | - |
| PASEF | 4 | 1.06 | 1.2 | 848.92 | 949.96 | - |
