# Supplementary material for: Democratized single-cell proteomics resolves cell state heterogeneity in skin tumors
Source: Life Sci Alliance. 2026 Jun 29;9(9):e202603759. doi: 10.26508/lsa.202603759 (PMC13315484; doi:10.26508/lsa.202603759)
Supplement: Reviewer comments [file LSA-2026-03759_review_history.pdf]

Democratized single-cell proteomics resolves cell state heterogeneity in skin tumors

Joseph Inns, Andrew Frey, Weng Ng, Matthias Trost, and Neil Rajan
DOI: https://doi.org/10.26508/lsa.202603759

Corresponding author(s): Joseph Inns, Newcastle University

Review Timeline:

Submission Date:	2026-05-07
Editorial Decision:	2026-06-02
Revision Received:	2026-06-08
Accepted:	2026-06-15

Scientific Editor: Tim Fessenden

Transaction Report:

(Note: With the exception of the correction of typographical or spelling errors that could be a source of ambiguity, letters and reports are not edited. The original formatting of letters and referee reports may not be reflected in this compilation.)

Please note that the manuscript was reviewed at *Review Commons* and these reports were taken into account in the decision-making process at *Life Science Alliance.*

Reviews

Review #1

This study presents a label-free single-cell proteomics (SCP) workflow designed to improve accessibility and reduce technical barriers for laboratories lacking specialized microfluidics or high-end instrumentation. The authors introduce an oil-immersion sample handling strategy combined with FACS-based single-cell isolation, LC-MS/MS analysis on a timsTOF HT platform, and neural-network-assisted spectral library generation. Applying this workflow to CYLD cutaneous syndrome (CCS) skin tumors, the authors analyze 419 individual tumor-derived cells, identifying ~700 proteins per cell on average and over 4,000 proteins in total. The approach enables discrimination between keratinocytes and immune cells and identifies a tumor-associated macrophage (TAM)-like population enriched for CD163, MRC1, and CD209 markers, which is further supported by immunofluorescence staining of tumor sections. The authors propose that the oil-immersion workflow improves protein recovery, reduces sample loss, and provides a more accessible platform for SCP in clinical tissues.

1. *Limited novelty relative to existing SCP workflows*

While the authors present the method as a "democratised" SCP approach, the conceptual advance appears modest. Oil-based sample handling strategies to reduce adsorption losses have been described previously, and the manuscript benchmarks the workflow only against a C18 capture method rather than widely used SCP platforms. Without broader comparisons, it is difficult to assess whether the method provides meaningful improvements in sensitivity, reproducibility, or cost. In addition, the reported ~700 proteins per cell is relatively modest compared with current state-of-the-art SCP datasets, suggesting that the claim of a broadly enabling SCP platform may be overstated. The authors should more clearly position their workflow relative to existing SCP technologies and clarify the primary technical advance.

2. *Insufficient performance characterization*

The benchmarking of the proposed workflow remains incomplete. Several important performance metrics that are typically reported for single-cell proteomics methods are missing, including quantitative reproducibility across cells and plates, missing value structure and overall data completeness, dynamic range and quantitative accuracy, potential batch effects across plates, and comparison of proteome coverage with corresponding bulk or mini-bulk references. Reporting these metrics would substantially strengthen the evaluation of the method and allow readers to better assess its quantitative performance.

3. *Limited biological depth and sample size*

The biological conclusions are based on only two patient samples carrying CYLD mutations. Although 419 cells were analyzed, these originate from a very small number of individuals, raising the possibility that the observed TAM population reflects patient-specific variation. In addition, no comparisons are made with normal skin or other tumor types, making it difficult to assess the general relevance of the findings. The biological conclusions should therefore be presented more cautiously, and the identified macrophage population should be framed as a preliminary, hypothesis-generating observation rather than a definitive discovery.

The manuscript addresses an important challenge in single-cell proteomics: lowering technical barriers to performing SCP experiments on clinical samples. The proposed oil-immersion workflow is conceptually straightforward and may reduce sample loss during processing. The demonstration of SCP profiling directly from human tumor tissue is also valuable.

However, the technical advance appears incremental, and the study does not yet convincingly establish that the method substantially improves accessibility or performance relative to existing SCP workflows. Additionally, the biological findings are based on a limited number of patient samples and therefore remain preliminary.

Overall, the work provides a potentially useful methodological variation but currently lacks the level of technical

validation and biological depth needed to represent a major advance.

Review #2

The manuscript by Inns et al. presents a single cell proteomics analysis based on oil immersion. The oil-immersion method has been compared to the conventional C18 tip method, resulting in outperforming proteome coverage. The method has been further demonstrated for the application to real tumor sample to reveal cell heterogeneity.

Major:

1. I suggest the authors clarifying the main contribution of the work regarding technical innovations. While the workflow is built "upon the successful implementation of oil-immersion liquid handling by others" (as stated in line 67), the results demonstrate the comprehensive performance comparison between the oil-immersion and the conventional C18 tip method, which has not been reported in the previous publication. Is the main contribution method development and performance evaluation, or application to tumor samples and biological discovery? It is recommended to re-focus to convey a clearer message.

2. As the details of oil immersion have not been reported in the previous publication, it is necessary to provide more information. Does the oil influence the hydrophobic peptides in sample preparation? For the comparison between the oil-immersion and the conventional C18 tip method, statistics of peptide hydrophobicity and protein location (e.g., cross membrane) are necessary.

3. Is sample injection operation changed? How to avoid the injection needle touching on the solid oil?

4. Method information of bioinformatics analysis is insufficient. Only the package names were mentioned without the used functions and parameters.

Minor:

5. Related to Comment 1, oil-immersion is not mentioned in the current title and abstract.

The manuscript by Inns et al. presents a single cell proteomics analysis based on oil immersion. The oil-immersion method has been compared to the conventional C18 tip method, resulting in outperforming proteome coverage. The method has been further demonstrated for the application to real tumor sample to reveal cell heterogeneity.

Advance:
The manuscript presents a single cell proteomics workflow outperforming the conventional C18 tip method.

Audience:
The manuscript is attractive to scientists in the single cell proteomics community, including those who work on technical development and application.

Review #3

Summary:

This manuscript presents an accessible, label-free single-cell proteomics (SCP) workflow. The methodology combines FACS sorting with an oil-immersion cell handling technique in standard 96-well plates, coupled with diaPASEF analysis on a Bruker timsTOF HT. The authors benchmark their oil-immersion method against an existing "One-Tip" method (Ye et al., 2024), demonstrating higher protein identification and better biological discriminating ability. They apply their workflow to profile 419 cells from a CYLD cutaneous syndrome (CCS) skin tumor, successfully distinguishing tumor keratinocytes from myeloid cells. By clustering, they found a subpopulation of tumor-associated macrophages (TAMs) expressing CD163, MRC1, and CD209, and validated by immunofluorescence.

Major comments:

- The study draws two main conclusions: 1) the oil-immersion SCP workflow offers a robust methodological advance

and 2) a distinct TAM subpopulation is present in CCS tumors. They are generally convincing, but several points require further clarification and strengthening.

- For the methodology part, "accessibility" requires more comparing data with current research results, and "cost-effectiveness" are currently presented as definitive conclusions without supporting data. The authors should either provide quantitative evidence for these claims or qualify them as preliminary assessments based on their specific setup. Methodological superiority would be more compelling if benchmarked against a broader range of established methods (e.g., Bubis et al. 2025), rather than solely against One-Tip. The authors could either provide such a comparison if possible.

- The discovery of the TAM subcluster lacks analytical transparency, as the steps from immune cell re-clustering to marker identification are unclear. And the so-called validation by immunofluorescence showed CD68/CD163 co-localization, without other two markers MRC1 and CD209 from profiling result, only confirming the presence of CD163 positive macrophage rather than the multi-marker signature or its enrichment. Tumor marker should also be included when illustrating the "island". The authors could provide five-color imaging corresponding to the validation conclusion if possible.

Minor comments:

1. Addressing Potential Oil Contamination: The technical concern about potential hexadecane carryover during the centrifugation step (Supp. Fig. 2) needs to be addressed. The authors should state whether any evidence of contamination (e.g., unusual peaks, retention time shifts) was observed in their blanks or samples, confirming that it does not affect LC-MS/MS performance.

2. Figure 1E: The reason for the choice of E3 ubiquitin ligases for biological sensitivity is not clearly justified.

3. Figure 1F: By "the most highly variable proteins", the claim that the oil-immersion workflow captures more biologically relevant variation is insufficiently supported without any explanation and references for those proteins different in two method.

4. Figures 2B: The protein identification counts per single cell vary dramatically, ranging from a few hundred to nearly two thousand, with a mean of 696 proteins per cell. While the average depth is moderate by current SCP standards, the extreme instability in identification rates across individual cells raises concerns about the robustness and reproducibility of the workflow.

5. Figures 2C: The claim that differences in protein identification numbers reflect biological differences between cell types is not justified.

6. Figure 3A-B: The description of the SingleR cell-type annotation is too brief. Please expand the results text to briefly explain the reference dataset used with more detail.

7. Figure 3F: The immunofluorescence lacks a stromal marker, making the description of cells "interdigitating between tumor islands" unclear and visually unsubstantiated.

This work represents a technical advance in the field of single-cell proteomics. Its primary significance lies in presenting a simplified, robust workflow that leverages widely available equipment (FACS, standard centrifuges, LC-MS/MS) and avoids the need for specialized, often expensive microfluidic devices. By demonstrating its utility on a challenging clinical sample (a rare tumor) and making the analysis pipeline accessible by adapting standard scRNA-seq tools (Seurat, SingleR), the study provides a practical blueprint for labs wishing to enter the SCP field. The identification of a TAM population in CCS tumors is a clinical discovery advance for that specific rare disease.

The study addresses a key bottleneck in SCP, the accessibility, that has been highlighted in recent reviews (e.g., Orsburn, 2024). It builds upon the concept of oil-air droplet manipulation previously described (Ctortecka et al., 2024) and adapts it for a simple, plate-based workflow. It is directly compared to the "One-Tip" method (Ye et al., 2024), showing improvements. Furthermore, it contributes to the growing application using SCP to dissect tumor heterogeneity, here in the understudied context of CCS.

The findings will be of significant interest to a broad audience:

1. Method developers in the proteomics and single-cell fields.

2. Translational and clinical researchers, particularly those studying rare cancer diseases, who may lack access to advanced SCP platforms but wish to apply SCP to their tumor samples.

My expertise is in proteomics, mass spectrometry and single-cell analysis methodologies. I have sufficient expertise to evaluate all parts of this manuscript.

1. General Statements [optional]

We thank the reviewers for their comments and for their time in performing this review.

2. Point-by-point description of the revisions

This section is mandatory. Please insert a point-by-point reply describing the revisions that were already carried out and included in the transferred manuscript.

#	Comment	Response
	**Reviewer 1**	
**1**	1. Limited novelty relative to existing SCP workflows<br>While the authors present the method as a "democratised" SCP approach, the conceptual advance appears modest. Oil-based sample handling strategies to reduce adsorption losses have been described previously, and the manuscript benchmarks the workflow only against a C18 capture method rather than widely used SCP platforms. Without broader comparisons, it is difficult to assess whether the method provides meaningful improvements in sensitivity, reproducibility, or cost. In addition, the reported ~700 proteins per cell is relatively modest compared with current state-of-the-art	The primary technical advance of this "democratised" workflow is the achievement of robust single-cell protein quantification using standard 96-well plates and the Bruker timsTOF HT—a mass spectrometer that is increasingly common in core facilities—rather than requiring the latest generation instruments or bespoke microfluidics. While ~700 proteins per cell is a moderate depth, the methodology prioritises accessibility, cost-effectiveness, and the potential for increased throughput over absolute proteomic depth to enable broader adoption.<br><br>To our knowledge this study demonstrates the first successful implementation of flow sorting cells and oil-immersion cell handling to derive biologically meaningful SCP data from clinical tissue. We chose to compare this workflow against the C18 capture method as this is a workflow which can also be performed without additional microfluidics instruments or specialised consumables.<br><br>**Action:** Clarification

	SCP datasets, suggesting that the claim of a broadly enabling SCP platform may be overstated. The authors should more clearly position their workflow relative to existing SCP technologies and clarify the primary technical advance.	
**2**	2. Insufficient performance characterization<br>The benchmarking of the proposed workflow remains incomplete. Several important performance metrics that are typically reported for single-cell proteomics methods are missing, including quantitative reproducibility across cells and plates, missing value structure and overall data completeness, dynamic range and quantitative accuracy, potential batch effects across plates, and comparison of proteome coverage with corresponding bulk or mini-bulk references. Reporting these metrics would substantially strengthen the evaluation of the method and allow readers to better assess its quantitative performance.	We appreciate that clearly stating performance metrics is an integral part of reporting a novel methodology. We direct readers towards the performance metrics which have been performed as part of this study and which we feel sufficiently address performance characterisation:<br><br>• Quantitative reproducibility is supported by a mean coefficient of variation (CV) of <20% per protein across cells. Mean CV figures have been added to the text.<br>• Mean data completeness was 11.9% per cell from a total of 5850 proteins. This metric and the % cells per protein have been added as supplementary figure 3.<br>• Dynamic range and sensitivity are demonstrated through the identification of 23 E3 ubiquitin ligases in the oil-immersion workflow compared to only 11 in the C18 capture method.<br>• Comparison of proteome coverage with corresponding bulk or mini-bulk references has been performed and displayed in figure 2A.<br>• Quantitative accuracy is assessed through correlation analysis of cell markers that are known to be co-expressed (Figure 2E).<br>• Uniform manifold projection (UMAP) confirms no significant batch effects across separate 96-well plates (Supplementary Figure 4).<br>• Proteome coverage per cell alongside 'true blanks' (blanks with reagents), has been performed (Figure 2B), as well as median protein identifications per cell type (Figure 2C).<br>• Quantitative efficacy of the mass spectrometry method has been reported (https://doi.org/10.1101/2025.09.22.677725) and this reference is included in the text.<br><br>**Action:** Mean CV figures added to text, Supplementary figure 3 added with data completeness metrics, clarification.

**3**	3. Limited biological depth and sample size<br>The biological conclusions are based on only two patient samples carrying CYLD mutations. Although 419 cells were analyzed, these originate from a very small number of individuals, raising the possibility that the observed TAM population reflects patient-specific variation. In addition, no comparisons are made with normal skin or other tumor types, making it difficult to assess the general relevance of the findings. The biological conclusions should therefore be presented more cautiously, and the identified macrophage population should be framed as a preliminary, hypothesis-generating observation rather than a definitive discovery.	While in-depth SCP was performed on a single rare tumor sample, the identified **CD163+/CD68+ TAM population** was validated in additional patient samples using immunofluorescence, reducing the likelihood of patient-specific variation. Furthermore, the SCP data clusters show high concordance with existing scRNA-seq datasets for CCS, providing cross-modality validation (as shown in the UMAP below, not for publication). We integrated the SCP dataset with an scRNA-seq dataset containing libraries from three different CCS patient samples with different genotypes. Here we found concordance between the biology of SCP and scRNA-seq generated data, with SCP cells (green) integrating with the corresponding clusters in an integrated UMAP (keratinocytes and myeloid cells, as per FACS markers).<br>[Figure removed by editorial staff per authors' request]<br>The access to rare tumour material is limited making repeated SCP analyses challenging, hence we chose to leverage this sample type where we had already obtained datasets, to further validate this workflow and our findings.<br><br>**Action:** Clarification.
**4**	The manuscript addresses an important challenge in single-cell proteomics: lowering technical barriers to performing SCP experiments on clinical samples. The proposed oil-immersion workflow is conceptually straightforward and may	We have demonstrated a methodology that improves accessibility to SCP workflows as described in response 1 - this is the main technological advance in this manuscript.<br><br>Although the biological findings are derived from a single SCP analysis they have been validated in multiple patient samples through immunofluorescence and are concordant with existing scRNA-seq data sets - see response 3.

	reduce sample loss during processing. The demonstration of SCP profiling directly from human tumor tissue is also valuable. However, the technical advance appears incremental, and the study does not yet convincingly establish that the method substantially improves accessibility or performance relative to existing SCP workflows. Additionally, the biological findings are based on a limited number of patient samples and therefore remain preliminary.<br>Overall, the work provides a potentially useful methodological variation but currently lacks the level of technical validation and biological depth needed to represent a major advance.	We believe sufficient technical validation has been provided - see response 2 - and we have used this methodology to identify a biological characteristic of CCS tumour tissue which has been validated in other samples and methodologies.<br><br>**Action:** Clarification.
**Reviewer 2**		
**5**	1. I suggest the authors clarifying the main contribution of the work regarding technical innovations. While the workflow is built "upon the successful implementation of oil-immersion liquid handling by others" (as stated in line 67), the results demonstrate the comprehensive performance comparison between the oil-immersion and the conventional C18 tip method, which has not been reported in the previous publication. Is the main contribution method development and performance evaluation, or application to tumor samples and biological discovery? It is recommended to re-focus to convey a clearer message.	The main contribution is the **technical evaluation and application** of an oil-immersion SCP workflow to clinical tissue to reveal functional protein-level heterogeneity that transcriptomics might miss. This is evidenced first, by the direct comparison showing the oil-immersion method's superiority over conventional C18 capture in identifying lineage-specific markers, and then through detailed cell type annotation and biological validation to demonstrate the biological faithfulness of the methodology.<br><br>We believe this is captured in the discussion where we state: *"We demonstrate the application of a novel oil-immersion SCP workflow coupled with an informatic pipeline in a skin tumor sample and demonstrate its utility in delineating tumour cell heterogeneity and identification of cell populations which may facilitate tumorigenesis. Our accessible workflow demonstrates superior biological sensitivity and cell type resolution to an established approach[14], without requiring specialised microfluidic instruments nor the latest generation mass spectrometer, facilitating broader adoption of SCP profiling."*

		**Action:** Clarification.
**6**	2. As the details of oil immersion have not been reported in the previous publication, it is necessary to provide more information. Does the oil influence the hydrophobic peptides in sample preparation? For the comparison between the oil-immersion and the conventional C18 tip method, statistics of peptide hydrophobicity and protein location (e.g., cross membrane) are necessary.	The use of oil is utilised in other publications (PMID 35817413, 40367333) and this approach has also been used in the cellenONE platform (PMID: 38977691). We agree that the points the reviewer raises are important, and propose that these should be addressed in future studies designed to compare, for example analysis of peptide hydrophobicity and membrane protein recovery.<br><br>**Action:** Clarification.
**7**	3. Is sample injection operation changed? How to avoid the injection needle touching on the solid oil?	No, the sample injection operation is unchanged.<br><br>The Evosep tips function as a sample clean up step. The solidified oil is retained in the 96-well plate, whilst only the peptide solution is loaded onto Evotip resin, hence the EvoSep LC system runs in its standard configuration.<br><br>**Action:** Clarification.
**8**	4. Method information of bioinformatics analysis is insufficient. Only the package names were mentioned without the used functions and parameters.	Statistical analysis and normalization were performed using Seurat v5.0, and cell type annotation utilized SingleR with the Human primary cell atlas as a reference. We use the standard analysis pipeline provide by these publications. Specific parameters for cell filtering and FDR (1%) are detailed in the Methods section.<br><br>The Seurat object is created from the DIA-NN protein groups output using the CreateSeuratObject() function.<br><br>**Action:** Specific reference to CreateSeuratObject() function and cell protein ID filtering added to methods, updated text to include reference to human primary cell atlas, and clarification.
**9**	5. Related to Comment 1, oil-immersion is not mentioned in the current title and abstract.	A reference to oil-immersion cell handling is included in the abstract and the graphical abstract.

		**Action:** Clarification.
	**Reviewer 3**	
**10**	The study draws two main conclusions: 1) the oil-immersion SCP workflow offers a robust methodological advance and 2) a distinct TAM subpopulation is present in CCS tumors. They are generally convincing, but several points require further clarification and strengthening.<br><br>For the methodology part, "accessibility" requires more comparing data with current research results, and "cost-effectiveness" are currently presented as definitive conclusions without supporting data.<br><br>The authors should either provide quantitative evidence for these claims or qualify them as preliminary assessments based on their specific setup.<br><br>Methodological superiority would be more compelling if benchmarked against a broader range of established methods (e.g., Bubis et al. 2025), rather than solely against One-Tip. The authors could either provide such a comparison if possible.	We summarise that this methodology provides increased accessibility primarily due to the instrumentation and consumable needs. For the instrumentation the workflow requires a FACS cell sorter such as the BD FACS Aria cell sorter, this type of instrument is common place in many Universities and research institutions, and we estimate is more prevalent than specialised microfluidics instruments such as the Cellenion cellenONE. It is beyond the scope of this study to assess the exact numbers of each instrument in each institution to perform a quantitative assessment, but we believe it is a generally accepted assumption that researchers are more likely to have access to a FACS sorter than a specialised microfluidics instrument. Additionally the use of a FACS instrument forgoes the need to use specialised plastic ware for cell collection such as the Cellenion proteochip, which also has an increased cost compared to a standard 96-well PCR plate used in this study.<br><br>In terms of cost we highlight that instrumentation that is likely pre-existing at an institution can be utilised, thereby reducing the cost-barrier and accessibility of this approach.<br><br>In this study we demonstrate the workflow on a more-affordable current generation instrument designed for high-throughput (Bruker timsTOF HT), which has a reduced list price compared to recently published SCP studies, utilising the Thermo Astral and Bruker timsTOF SCP, thereby reducing cost.<br><br>Many current SCP platforms rely on proprietary microfluidic chips (e.g. proteoCHIP, SciCHIP) which can cost $100+ per plate. Our oil-immersion method utilises standard 96-well PCR plates (approx. $0.1 per sample) and bulk purchased hexadecane, reducing the consumable cost per cell to a negligible fraction of specialised alternatives.<br><br>Additionally we assess that this methodology would result in a reduced cost per cell at < $10/cell for digestion. As reported by Smythers and Orsburn 2024., the mean cost per cell for label free SCP is $15.04/cell, and trending towards increased cost. By using the calculator provided by

		Smythers and Orsburn 2024, we calculate our methodology would result in an estimated cost of $9.83/cell.<br><br>We acknowledge that Bubis et al. (2025) achieved an unprecedented depth of ~5,300 proteins. However, our workflow does not aim to match this level of resolution and instead demonstrates that in a "democratised" workflow, a depth of ~700 proteins/cell is sufficient to resolve clinically relevant biology, such as the CD163+/CD68+ TAM population in rare CCS tumors<br><br>**Action:** Clarification.
**11**	The discovery of the TAM subcluster lacks analytical transparency, as the steps from immune cell re-clustering to marker identification are unclear. And the so-called validation by immunofluorescence showed CD68/CD163 co-localization, without other two markers MRC1 and CD209 from profiling result, only confirming the presence of CD163 positive macrophage rather than the multi-marker signature or its enrichment. Tumor marker should also be included when illustrating the "island". The authors could provide five-color imaging corresponding to the validation conclusion if possible.	We appreciate the reviewer's request for greater transparency regarding our computational pipeline and histological validation.<br><br>1. Analytical Transparency: To identify the TAM subcluster, we employed a hierarchical sub-clustering strategy. After initial broad annotation using SingleR , we isolated the 196 immune cells (dendritic cells, macrophages, and monocytes) for re-clustering in Seurat. Cluster-specific markers were identified using the Wilcoxon rank-sum test with a significance threshold of an adjusted p-value < 0.05 and a log2 fold-change > 1. This process revealed Cluster 2 as a distinct population defined by a 94-protein signature, including CD163, MRC1, and CD209.<br><br>2. Histological Validation: While our proteomic profiling identified a multi-marker signature, we prioritized CD163 for immunofluorescence (IF) validation as it is a well-established and robust surrogate for the M2 immunosuppressive phenotype in skin tumors. We acknowledge that five-color imaging would provide a more comprehensive validation of the co-expression of MRC1 and CD209; however, the co-localization of CD68 and CD163 in the tumor stroma provides strong evidence for the presence of the identified TAM population. To strengthen this case we have performed additional immunofluorescence experiments with CCS tumor sections where we have used CD68/CD163/CD206 to show co-localisation of all 3 markers in stroma localised cells (below and now in figure 3F).

		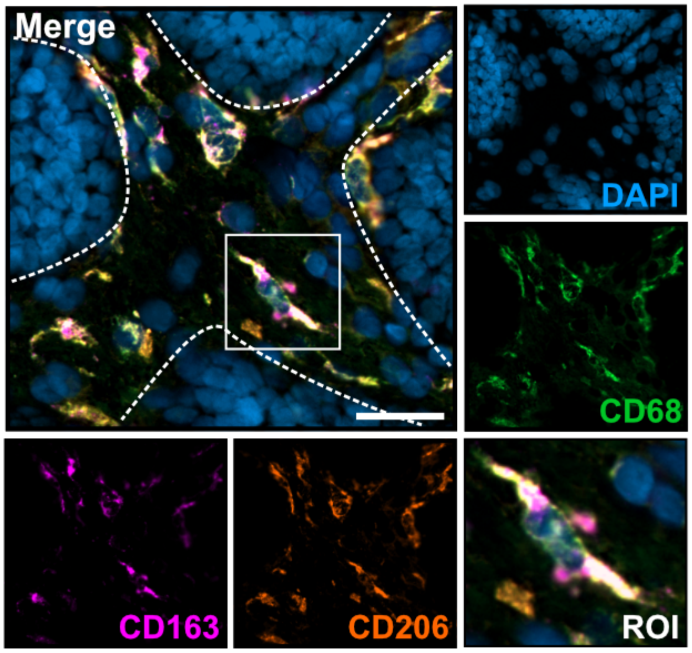<br><br><br>3. Delineating Tumor "Islands": In Figure 3F, tumor "islands" are delineated by dense clusters of DAPI-stained nuclei. These high-density epithelial structures are morphologically distinct from the surrounding stroma. We have updated the figure legend to explicitly state how nuclear density (DAPI) is used to identify these tumor islands. Furthermore To address the lack of a stromal marker, we describe CD68/CD163/CD206 positive cells "interdigitating" between tumor islands, which is visually substantiated by the large, distinct clusters of DAPI-stained tumor nuclei (islands) separated by the stromal space. This structure of tumor islands of tightly organised cells surrounded by stroma has been published previously for CCS tumors (https://www.ncbi.nlm.nih.gov/books/NBK555820/).<br><br>**Action:** Additional immunofluorescence images with MRC1 (CD206) included in figure 3, text added to main text and figure legend to clarify CCS tumor histology and marked on the figure to aid interpretation, clarification.
**12**	1. Addressing Potential Oil Contamination: The technical concern about potential hexadecane carryover during the centrifugation step (Supp. Fig. 2) needs to be addressed. The authors should state whether any evidence of contamination (e.g., unusual	Hexadecane is a saturated alkane and ionizes poorly by ESI in positive mode. Additionally, it has no basic sites, no heteroatoms, and no easily protonated groups so it is not typically seen in a proteomics ESI run, making it suitable for SCP analysis. We have analysed spectra in “blank” data which has the reaction mixture in contact with hexadecane oil (below), and find that the signal is either noise, not present, or does not follow the pattern we would expect for contamination (hydrocarbon ladder and late eluting peak).

<table>
<tr>
<td></td>
<td>peaks, retention time shifts) was observed in their blanks or samples, confirming that it does not affect LC-MS/MS performance.</td>
<td>Biological samples such as subcutaneous fat may also contain lipids and oil that will be carried over(such as palmitic acid, a common environmental contaminant which we detect here), and we expect that the evotip has a role in mitigating such an effect during cleanup or retention during injection. We did not see any effects of contamination in our analysis that impacted on peptides detected and biological findings that were validated using orthogonal technologies, however we agree that this is a potential aspect to consider in future studies.

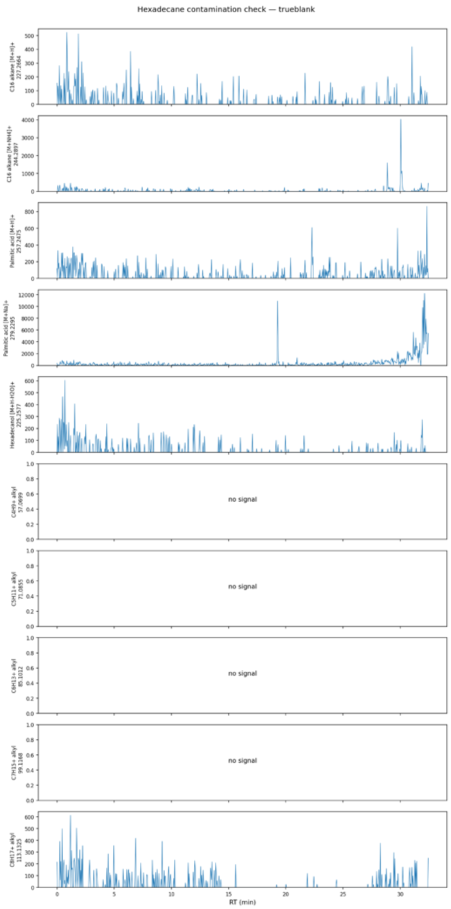

With regards to contamination and impacts on retention, profiles of base peak chromatograms from bulk samples 500 runs apart are similar in appearance, and dominant peaks only display minor drift (under 30 seconds, below). Furthermore, indexed retention correlates closely with retention time, suggesting that any impact of elution time drifting is minor.</td>
</tr>
</table>

		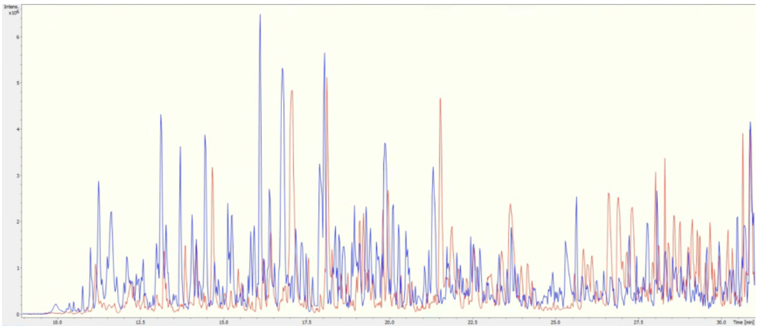 Base Peak chromatograms of a bulk analysis from plate 1 (red) and plate 6 (blue).<br><br>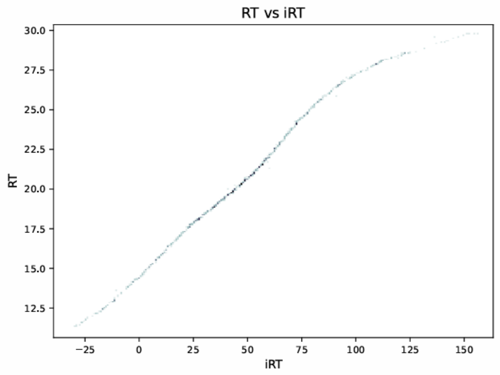<br><br>Precursor retention time (mins) against indexed retention time (scaled from min –25, max 150). Figure assesses precursor retention times from the last single cell to be analysed.<br><br>**Action:** Clarification.
**13**	2. Figure 1E: The reason for the choice of E3 ubiquitin ligases for biological sensitivity is not clearly justified.	E3 ubiquitin ligases were chosen to assess workflow sensitivity as they are detected with a high dynamic range within our dataset and others, are of biological importance, and have previously been used as a standard for assessing SCP workflow sensitivity (Ctortecka et al. 2024, ttps://doi.org/10.1038/s41467-024-49651-w)<br><br>**Action:** Justification.
**14**	3. Figure 1F: By "the most highly variable proteins", the claim that the oil-immersion workflow captures more biologically relevant variation is insufficiently supported without any explanation and	We calculated protein variability using the standardised workflow in Seurat and the FindVariableFeatures() function. We list the top20 from each methodology and use the ACT webtool to identify if any of the top 20 variable proteins are Keratinocyte markers or DEGs or myeloid cell markers.

	references for those proteins different in two method.	A reference to the web tool used is now added in the text. https://link.springer.com/article/10.1186/s13073-023-01249-5<br><br>The 20 most variable proteins were analyzed because they are typically enriched for markers that distinguish distinct cell lineages. In the oil-immersion workflow, these proteins successfully identified 10 keratinocyte and 7 myeloid markers, compared to only 3 and 5 in the C18 workflow.<br><br>**Action:** Reference added, clarification.
**15**	4. Figures 2B: The protein identification counts per single cell vary dramatically, ranging from a few hundred to nearly two thousand, with a mean of 696 proteins per cell. While the average depth is moderate by current SCP standards, the extreme instability in identification rates across individual cells raises concerns about the robustness and reproducibility of the workflow.	We direct to previous studies where a similar range in the number of protein identifications has been observed (https://doi.org/10.1038/s41467-024-49651-w , https://doi.org/10.1038/s41467-025-67367-3). We believe part of the variation in protein group identifications is due to cell to cell heterogeneity, however we can not rule out that this may also in part be due to technical differences in sample processing.<br><br>In our experimental design we suggest that the variation in protein IDs (averaging 696) may reflect inherent biological differences between cell types, with larger, more metabolically active CD45+/CD74+ immune cells yielding significantly more proteins (mean 914) than tumor keratinocytes (mean 467). Additionally, cell samples with a large dynamic range (i.e high abundance of keratins) may hamper the detection of lower abundance proteins.<br><br>We have performed analyses to measure the robustness of this workflow, including protein CV/cell, protein marker correlation, blank detection quantification, and biological clustering (figure 2) which display the robustness of the workflow.<br><br>**Action:** Clarification.
**16**	5. Figures 2C: The claim that differences in protein identification numbers reflect biological differences between cell types is not justified.	We appreciate sufficient evidence is not provided to fully substantiate this claim therefore we have modified the text to read: *“The number of protein identifications differed in the FACS sorted cell groups, with a mean of 467 and 914 proteins per cell in CD45-/CD200+ and CD45+/CD74+ cells respectively (Figure 2C), which may represent biological differences between these two cell types.”*<br><br>**Action:** Text edited.

**17**	6. Figure 3A-B: The description of the SingleR cell-type annotation is too brief. Please expand the results text to briefly explain the reference dataset used with more detail.	We utilised the celldex package and the Human Primary cell Atlas (HPCA) database within the SingleR framework to provide unbiased, reference based annotation of the 419 analysed cells. The use of celldex package is now referenced in the results text along with the use of the HPCA - these are both referenced:<br>SingleR and celldex:<br>https://www.nature.com/articles/s41590-018-0276-y<br>HPCA:https://link.springer.com/article/10.1186/1471-2164-14-632<br><br>**Action:** Text added and referenced.
**18**	7. Figure 3F: The immunofluorescence lacks a stromal marker, making the description of cells "interdigitating between tumor islands" unclear and visually unsubstantiated.	Also addressed in point 11. To address the lack of a stromal marker, we describe CD68/CD163/CD206 positive cells "interdigitating" between tumor islands, which is visually substantiated by the large, distinct clusters of DAPI-stained tumor nuclei (islands) separated by the stromal space. This structure of tumor islands of tightly organised cells surrounded by stroma has been published previously for CCS tumors.<br>(https://www.ncbi.nlm.nih.gov/books/NBK555820/).<br><br>**Action:** Additional immunofluorescence images with MRC1 (CD206) included in figure 3, text added to main text and figure legend to clarify CCS tumor histology and marked on the figure to aid interpretation, clarification.

June 2, 2026

RE: Life Science Alliance Manuscript #LSA-2026-03759

Joseph Inns
Newcastle University
Translational and clinical research institute
Unknown
United Kingdom [GB]

Dear Dr. Inns,

Thank you for submitting your revised manuscript entitled "Democratised single cell proteomics uncovers a tumor associated macrophage signature in CYLD cutaneous syndrome skin tumors". As you will see, the reviewers are satisfied with no further requests. We would be happy to publish your paper in Life Science Alliance pending final revisions necessary to meet our formatting guidelines.

MANUSCRIPT ORGANIZATION AND FORMATTING:

To avoid unnecessary delays in the acceptance and publication of your paper, please read the following information carefully. Full guidelines are available on our Instructions for Authors page, https://www.life-science-alliance.org/authors

Submission of a paper that does not conform to Life Science Alliance guidelines will delay the acceptance of your manuscript.

-Please upload your main manuscript text as an editable doc file.
-Please upload all figure files as individual ones, including the supplementary figure files; all figure legends should only appear in the main manuscript file
-Please add a Running Title in our system.
-Please add a Summary Blurb/Alternate Abstract and a Category for your manuscript in our system.
-Please add the X and Bluesky handles of your host institute/organization, as well as your own, and/or one of the authors, in our system.
-Please consult our manuscript preparation guidelines https://www.life-science-alliance.org/manuscript-prep and make sure your manuscript sections are in the correct order.
-Please remove the Graphical Abstract from the manuscript text and upload it separately with the file designation "Graphical Abstract."
-Please add your main, supplementary figure, and table legends to the main manuscript text after the references section.
-It is recommended to exclude figures from the manuscript text and upload them separately.
-Please upload your Tables in editable .doc or Excel format.
-Please add an Author Contributions section to your main manuscript text and in the system for each author.
-Please add details to the Methods section on FACS sorting (instrumentation, preparation, antibodies) and immunofluorescence imaging (objectives, illumination, acquisition software, processing).
-Please be sure to update the Data Availability statement and ensure the deposited data are available without the reviewer accession token.

We encourage our authors to provide original source data, particularly uncropped/-processed electrophoretic blots and spreadsheets for the main figures of the manuscript. If you would like to add source data, we would welcome one PDF/Excel-file per figure for this information. These files will be linked as supplementary "Source Data" files.

It is Life Science Alliance policy that if requested, original data images must be made available to the editors. Failure to provide original images upon request will result in unavoidable delays in publication. Please ensure that you have access to all original data images prior to final submission.

If you are planning a press release on your work, please inform us immediately to allow informing our production team and scheduling a release date.

We welcome submissions of potential cover images for the issue of LSA in which your work would appear. If you have high quality images associated with this work, please feel free to email these, with a caption, to the journal office.

LSA encourages authors to provide a 30-60 second video where the study is briefly explained. These videos will be appear embedded with the manuscript online at Life Science Alliance, and on social media to promote the published paper and authors

(for examples, see https://docs.google.com/document/d/1-UWCfbE4pGcDdcgzcmiuJl2XMBJnxKYeqRvLLrLSo8s/edit?usp=sharing). Corresponding or first-authors are welcome to submit the video. Please submit only one video per manuscript. The video can be emailed to contact@life-science-alliance.org

FINAL FILES:

To upload the final version of your manuscript, please log in to your account: https://lsa.msubmit.net/cgi-bin/main.plex
You will be guided to complete the submission of your revised manuscript and to fill in all necessary information. Please get in touch in case you do not know or remember your login name.

The following items are required for acceptance.

-- An editable version of the final text (.DOC or .DOCX) is needed for copyediting (no PDFs).

-- High-resolution figure, supplementary figure and video files uploaded as individual files: See our detailed guidelines for preparing your production-ready images, https://www.life-science-alliance.org/authors

-- Summary blurb (enter in submission system): A short text summarizing in a single sentence the study (max. 200 characters including spaces). This text is used in conjunction with the titles of papers, hence should be informative and complementary to the title. It should describe the context and significance of the findings for a general readership; it should be written in the present tense and refer to the work in the third person. Author names should not be mentioned.

The license to publish form must be signed before your manuscript can be sent to production. A link to the license to publish form will be available to the corresponding author only. Please take a moment to check your funder requirements.

Reviews, decision letters, and point-by-point responses associated with peer-review at Life Science Alliance will be published, alongside the manuscript. If you do want to opt out of having the reviewer reports and your point-by-point responses displayed, please let us know immediately.

Thank you for your attention to these final processing requirements. Please revise and format the manuscript and upload materials as soon as you are able.

Thank you for this interesting contribution to the literature. We look forward to publishing your paper in Life Science Alliance.

Sincerely,

Tim Fessenden
Scientific Editor
Life Science Alliance

Reviewer #1 (Comments to the Authors (Required)):

The authors have made a constructive revision and have addressed the major concerns raised during the first round. The revised manuscript now provides clearer positioning of the oil-immersion workflow, additional performance metrics including mean CV, data completeness, blank measurements and plate-level batch assessment, a more detailed description of the Seurat/SingleR-based analysis, a PRIDE accession, and additional immunofluorescence evidence including CD68/CD163/CD206 staining. These changes substantially improve the transparency of the work and make the technical and biological claims easier to evaluate.
Overall, I think the manuscript has improved substantially and is now suitable for publication in Life Science Alliance. The study presents a practical and accessible adaptation of single-cell proteomics for clinical tissue samples, and the revised data provide sufficient technical support for the main conclusions.

Reviewer #2 (Comments to the Authors (Required)):

The authors have addressed all my comments properly.

June 15, 2026

RE: Life Science Alliance Manuscript #LSA-2026-03759R

Dr. Joseph Inns
Newcastle University
Translational and clinical research institute
Newcastle upon Tyne NE2 4HH
United Kingdom

Dear Dr. Inns,

Thank you for submitting your Research Article entitled "Democratized single-cell proteomics resolves cell state heterogeneity in skin tumors". It is a pleasure to let you know that your manuscript is now accepted for publication in Life Science Alliance. Congratulations on this interesting work.

The final published version of your manuscript will be deposited by us to PubMed Central upon publication.

Your manuscript will now progress through copyediting and proofing. It is journal policy that authors provide original data upon request.

Your article will publish open access upon publication under a CC-BY license.

Reviews, decision letters, and point-by-point responses associated with peer-review at Life Science Alliance will be published, alongside the manuscript. If you do want to opt out of having the reviewer reports and your point-by-point responses displayed, please let us know immediately.

IMPORTANT: If you will be unreachable at any time, please provide us with the email address of an alternate author. Failure to respond to routine queries may lead to unavoidable delays in publication.

Scheduling details will be available from our production department. You will receive proofs shortly before the publication date. Only essential corrections can be made at the proof stage so if there are any minor final changes you wish to make to the manuscript, please let the journal office know now.

DISTRIBUTION OF MATERIALS:
Authors are required to distribute freely any materials used in experiments published in Life Science Alliance. Authors are encouraged to deposit materials used in their studies to the appropriate repositories for distribution to researchers.

You can contact the journal office with any questions, contact@life-science-alliance.org

Again, congratulations on a very nice paper. I hope you found the review process to be constructive and are pleased with how the manuscript was handled editorially. We look forward to future exciting submissions from your lab.

Sincerely,

Tim Fessenden
Scientific Editor
Life Science Alliance
